# Peripheral blood markers predict immunotherapeutic efficacy in patients with advanced non-small cell lung cancer: A multicenter study

**DOI:** 10.3389/fgene.2022.1016085

**Published:** 2022-10-20

**Authors:** Shuai Liu, Liuyuan Zhao, Guohua Zhou

**Affiliations:** ^1^ Department of Anesthesiology, Affiliated Drum Tower Hospital, Medical School of Nanjing University, Nanjing, China; ^2^ Department of Pain Medicine, Harbin Medical University Cancer Hospital, Harbin, China; ^3^ Department of Anesthesiology, Ningbo First Hospital, Zhejiang, China; ^4^ Department of Anesthesiology, The First Affiliated Hospital of Dalian Medical University, Dalian, China

**Keywords:** non-small cell lung cancer, biomarker, peripheral blood, neutrophil-to-lymphocyte ratio, red blood cell distribution width

## Abstract

This study aims to investigate the prognostic impact of peripheral blood markers in patients with advanced non-small cell lung cancer (NSCLC) undergoing immunotherapy. In the current multicenter study, 157 advanced NSCLC cases treated by immunotherapy at three institutions were included. Biochemical parameters in baseline peripheral blood were collected. The associations between biochemical parameters and prognosis were investigated by the Kaplan–Meier survival analyses and Cox regression, and the predictive performances of biomarkers were evaluated *via* receiver operating characteristic analysis. The neutrophil-to-lymphocyte ratio (NLR) (progression-free survival [PFS]: hazard ratio [HR], 1.766; 95% confidence interval [CI], 1.311–2.380; *p* < 0.001; overall survival [OS]: HR, 1.283; 95% CI, 1.120–1.469; *p* < 0.001) and red blood cell distribution width (RDW) (PFS: HR, 1.052; 95% CI, 1.005–1.102; *p* = 0.031; OS: HR, 1.044; 95% CI, 1.001–1.091; *p* = 0.042) were revealed as independent predictors for both PFS and OS. In addition, NLR ≥3.79 (1-year PFS, 24.2% [95% CI, 15.2%–38.4%] *versus* 27.3% [95% CI, 18.2%–41.1%], *p* = 0.041; 1-year OS, 44.2% [95% CI, 32.5%–60.1%] *versus* 71.8% [95% CI, 60.6%–85.2%], *p* < 0.001) or RDW ≥44.8 g/L (1-year PFS, 19.2% [95% CI, 11.4%–32.3%] *versus* 31.7% [95% CI, 21.9%–46.0%], *p* = 0.049; 1-year OS, 54.0% [95% CI, 42.7%–68.3%] *versus* 63.1% [95% CI, 50.6%–78.6%], *p* = 0.014) was significantly correlated to poorer PFS and OS than NLR < 3.79 or RDW <44.8 g/L. Moreover, NLR and RDW achieved areas under the curve with 0.651 (95% CI, 0.559–0.743) and 0.626 (95% CI, 0.520–0.732) for predicting PFS, and 0.660 (95% CI, 0.567–0.754) and 0.645 (95% CI, 0.552–0.739), for OS. Therefore, PLR and RDW could help predict the immunotherapeutic efficacy of advanced NSCLC.

## Introduction

Immune checkpoint inhibitors (ICIs), which target programmed cell death 1 (PD-1) and its ligand (PD-L1), are capable of inducing sustained antitumor effects, ushering in the therapeutic era for multiple malignant neoplasms (Okazaki et al., 2013; Ribas and Wolchok, 2018). In spite of this significant breakthrough, potent immunotherapeutic responses were only observed in approximately 20% advanced non-small cell lung cancer (NSCLC) population (Borghaei et al., 2015; Brahmer et al., 2015; Reck et al., 2016). Hence, precise recognition of patients who have the potential to derive additional benefits from ICIs is essential for the personalized treatment of advanced NSCLC.

Several biomarkers for immunotherapeutic efficacy of advanced NSCLC, such as tumor mutation burden (TMB), PD-L1, and tumor-infiltrating lymphocytes, have been revealed in previous publications (Kerr et al., 2015; Meng et al., 2015; High TMB Predicts Immunotherapy Benefit, 2018). However, in the current clinical practice, recognizing these signatures primarily resorts to core biopsy, which is unable to quantify the whole heterogeneity of tumors attributable to the limited specimens and simultaneously brings about a significant morbidity risk considering its invasive manipulation (Kerr et al., 2015; McLaughlin et al., 2016). As a result, a reliable and noninvasive instrument to predict the immunotherapeutic efficacy of advanced NSCLC is urgently needed.

Previous studies indicated that tumor-related inflammation played an important role in regulating tumor progression and immune infiltration (Jomrich et al., 2021). Moreover, biochemical parameters in peripheral blood provide a convenient and cost-effective path for reflecting the inflammatory status and their predictive potentials for immunotherapeutic efficacy have been investigated in various types of cancers receiving ICIs (Fukui et al., 2019; Nenclares et al., 2021; Valero et al., 2021). However, pieces of evidence for the value of biochemical parameters in peripheral blood in advanced NSCLC are insufficient. Therefore, this study, based on a multicenter population, proposes to explore the associations between pretreatment peripheral blood markers and prognosis in advanced NSCLC populations treated with ICIs.

## Materials and methods

### Study population

**FIGURE 1:**
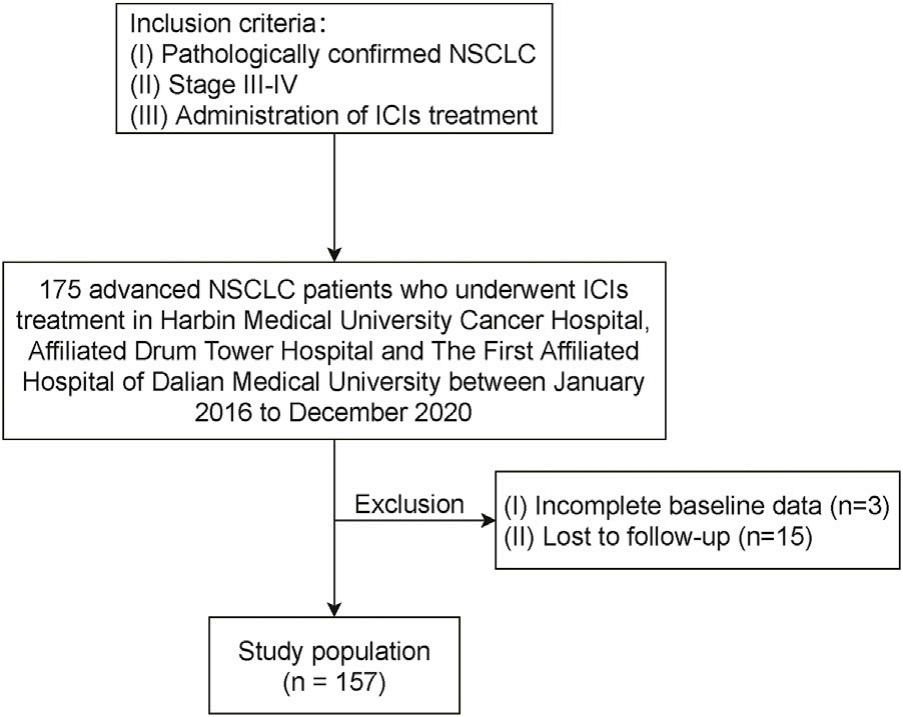
Flow chart illustrating patient inclusion. NSCLC, non-small cell lung cancer; ICI, immune checkpoint inhibitor.

Approval of the institutional review boards and ethics committees of Harbin Medical University Cancer Hospital, Affiliated Drum Tower Hospital, and The First Affiliated Hospital of Dalian Medical University and a waiver for informed consent were obtained. Consecutive advanced NSCLC patients who underwent ICIs treatment in the abovementioned institutions between January 2016 to December 2020 were reviewed (Figure 1). Patients were included in this study when meeting the following criteria: 1) pathologically confirmed NSCLC; 2) stage III–IV; 3) administration of treatment ICIs, regardless of pretreatment line. The exclusion criteria included incomplete baseline data and lost to follow-up. All patients completed the follow-up survey before August 2022.

### Data collection

Clinicopathologic information was collected from electronic medical systems. Follow-up data were obtained through outpatient visits and telephone surveys. Overall survival (OS) was determined as the interval from initial ICI treatment to death or last follow-up. Progression-free survival (PFS) was calculated as the duration between initial ICI treatment and disease progress, death, or last follow-up.

Baseline peripheral blood samples were acquired within 7 days before immunotherapy, and routine blood biochemical parameters were collected. The inflammatory indexes were obtained based on the following formula: platelet-to-lymphocyte ratio (PLR) and absolute platelet count/absolute lymphocyte count; neutrophil-to-lymphocyte ratio (NLR) and absolute neutrophil count/absolute lymphocyte count; derived NLR (dNLR) and absolute neutrophil count/(white blood cell count-absolute neutrophil count); monocyte-to-lymphocyte ratio (MLR) and absolute monocyte count/absolute lymphocyte count; and systemic immune-inflammation index (SII), absolute neutrophil count×absolute platelet count/absolute lymphocyte count.

### Statistical analysis

Pearson’s chi-squared test and Student’s *t*-test were implemented to compare the categorical and continuous parameters, respectively. Cox regressions and Kaplan–Meier survival analyses were conducted to recognize predictors for OS and PFS *via* the backward stepwise selection. The abovementioned statistical analyses were done using SPSS (version 23.0, IBM, Armonk, NY, United States) R software (version 4.1.1, http://www.R-project.org). A *p* value less than 0.05 was considered statistically significant.

## Results

### Clinicopathologic characteristics

The clinicopathologic characteristics were displayed in Table 1. The entire cohort included 97 (61.78%) men and 60 (38.22%) women, and the mean age for the whole population was 60.82 years. Smoking history was identified in 95 (60.51%) patients. ECOG PS 1 (*n* = 136, 86.62%) accounted for the largest proportion. Most patients were diagnosed as stage Ⅳ (*n* = 134, 85.35%) and squamous cell carcinoma (*n* = 94, 59.87%). Regarding the peripheral blood indexes, the mean level of PLR, NLR, dNLR, MLR, SII, hemoglobin (HGB), red blood cell count (RBC), white blood cell count (WBC), percentage of neutrophil (NEUT%), percentage of lymphocyte (LYM%), percentage of monocyte (MONO), percentage of eosinophils (EOS), percentage of basophil granulocytes (BASO), platelet (PLT), hematocrit (HCT), mean corpuscular volume (MCV), mean corpuscular hemoglobin (MCH), mean corpuscular hemoglobin concentration (MCHC), red blood cell distribution width (RDW), plateletcrit (PCT), platelet distribution width (PDW), and platelet-larger cell ratio (P-LCR) were 194.97, 4.47, 2.75, 0.47, 1,182.14, 120.25 g/L, 4.11 × 10^12/L, 8.35 × 10^9/L, 68.55%, 20.28%, 8.08%, 2.36%, 0.29%, 263.72 × 10^9/L, 36.96%, 90.26 fL, 29.34 ng, 324.82 g/L, 46.10 g/L, 26.01%, 11.18 fL and 24.39%. In addition, in subgroup analyses between 117 patients evaluated as progressive disease (PD) or stable disease (SD) and 40 patients with partial response (PR) or complete response (CR), patients classified as PR or CR were associated with a significantly higher proportion of smoking history (75% *versus* 55.56%, *p* = 0.030) and a higher level of HCT (38.41% *versus* 36.47%, *p* = 0.047).

**TABLE 1:** Baseline characteristics of patients.

Characteristics	Entire cohort (*n* = 157)	PD + SD (*n* = 117)	PR + CR (*n* = 40)	*p* value
Age (years), mean ± SD	60.82 ± 10.45	60.59 ± 10.89	61.50 ± 9.16	0.636
Sex, *n* (%)				0.562
Male	97 (61.78)	71 (60.68)	25 (62.50)	
Female	60 (38.22)	46 (39.21)	15 (37.50)	
Smoking, *n* (%)				0.030
Ever	95 (60.51)	65 (55.56)	30 (75.00)	
Never	62 (39.49)	52 (44.44)	10 (25.00)	
ECOG PS, *n* (%)				0.551
0	12 (7.64)	7 (5.98)	5 (12.50)	
1	136 (86.62)	103 (88.03)	33 (82.50)	
2	8 (5.10)	6 (5.13)	2 (5.00)	
3	1 (0.64)	1 (0.85)	0 (0.00)	
Stage, *n* (%)				0.335
Ⅲ	23 (14.65)	38 (32.48)	11 (27.50)	
Ⅳ	134 (85.35)	67 (57.26)	27 (67.50)	
Histology, *n* (%)				0.431
Squamous cell carcinoma	94 (59.87)	67 (57.26)	27 (67.50)	
Adenocarcinoma	49 (31.21)	38 (32.48)	11 (27.50)	
Others	14 (8.90)	12 (10.26)	2 (5.00)	
Treatment line, *n* (%)				0.856
First	84 (53.50)	62 (53.00)	22 (55.00)	
Not first	73 (46.50)	55 (47.00)	18 (45.00)	
Peripheral blood index				
PLR, mean ± SD	194.97 ± 103.36	196.66 ± 100.63	190.00 ± 112.17	0.726
NLR, mean ± SD	4.47 ± 2.86	4.68 ± 3.03	3.85 ± 2.24	0.113
dNLR, mean ± SD	2.75 ± 1.74	2.80 ± 1.68	2.61 ± 1.93	0.546
MLR, mean ± SD	0.47 ± 0.31	0.49 ± 0.33	0.40 ± 0.22	0.112
SII, mean ± SD	1,182.14 ± 943.78	1,215.66 ± 1,000.62	1,084.06 ± 756.06	0.448
HGB (g/L), mean ± SD	120.25 ± 19.10	118.56 ± 19.51	125.21 ± 17.14	0.057
RBC (10^12/L), mean ± SD	4.11 ± 0.61	4.06 ± 0.62	4.24 ± 0.56	0.122
WBC (10^9/L), mean ± SD	8.35 ± 3.33	8.39 ± 3.56	8.25 ± 2.56	0.820
NEUT%, mean ± SD	68.55 ± 12.27	68.93 ± 12.58	67.46 ± 11.40	0.514
LYM%, mean ± SD	20.28 ± 9.24	19.70 ± 9.12	21.98 ± 9.50	0.179
MONO%, mean ± SD	8.08 ± 3.28	8.09 ± 3.20	8.06 ± 3.55	0.962
EOS%, mean ± SD	2.36 ± 2.86	2.40 ± 3.15	2.24 ± 1.79	0.755
BASO%, mean ± SD	0.29 ± 0.28	0.27 ± 0.19	0.34 ± 0.44	0.202
PLT (10^9/L), mean ± SD	263.72 ± 96.82	259.38 ± 103.41	276.43 ± 73.91	0.338
HCT (%), mean ± SD	36.96 ± 5.34	36.47 ± 5.44	38.41 ± 4.83	0.047
MCV (fL), mean ± SD	90.26 ± 5.42	90.03 ± 5.69	90.92 ± 4.52	0.369
MCH (pg), mean ± SD	29.34 ± 1.94	29.24 ± 2.04	29.62 ± 1.62	0.287
MCHC (g/L), mean ± SD	324.82 ± 12.23	324.55 ± 13.45	325.60 ± 7.71	0.640
RDW (g/L), mean ± SD	46.10 ± 5.94	46.55 ± 5.98	44.80 ± 5.73	0.110
PCT (%), mean ± SD	26.01 ± 9.21	25.50 ± 9.69	27.53 ± 7.56	0.230
PDW (fL), mean ± SD	11.18 ± 1.77	11.16 ± 1.86	11.23 ± 1.53	0.830
P-LCR (%), mean ± SD	24.39 ± 7.47	24.30 ± 7.56	24.65 ± 7.26	0.804

PD, progressive disease; SD, stable disease; PR, partial response; CR, complete response; ECOG PS, Eastern Cooperative Oncology Group Performance Status; PLR, platelet-to-lymphocyte ratio; NLR, neutrophil-to-lymphocyte ratio; MLR, monocyte-to-lymphocyte ratio; SII, systemic immune-inflammation index; HGB, hemoglobin; RBC, red blood cell count; WBC, white blood cell count; NEUT, neutrophil; LYM, lymphocyte; MONO, monocyte; EOS, eosinophils; BASO, basophil granulocytes; PLT, platelet; HCT, hematocrit; MCV, mean corpuscular volume; MCH, mean corpuscular hemoglobin; MCHC, mean corpuscular hemoglobin concentration; RDW, red blood cell distribution width; PCT, plateletcrit; PDW, platelet distribution width; P-LCR, platelet-larger cell ratio; SD, standard deviation.

### Prognostic impact of peripheral blood markers

In the Cox survival analyses (Table 2), smoking history (hazard ratio [HR], 0.457; 95% confidence interval [CI], 0.306–0.683; *p* < 0.001), ECOG PS ≥ 1 (HR, 3.040; 95% CI, 1.386–6.668; *p* = 0.006), stage Ⅳ (HR, 0.465; 95% CI, 0.267–0.812; *p* = 0.007), NLR (HR, 1.766; 95% CI, 1.311–2.380; *p* < 0.001), dNLR (HR, 0.489; 95% CI, 0.321–0.744; *p* = 0.001), MLR (HR, 0.203; 95% CI, 0.044–0.929; *p* = 0.040), HGB (HR, 0.002; 95% CI, 0.001–0.171; *p* = 0.010), HCT (HR, 2.220; 95% CI, 1.183–4.166; *p* = 0.013), MCV (HR, 0.678; 95% CI, 0.525–0.876; *p* = 0.003), and RDW (HR, 1.052; 95% CI, 1.005–1.102; *p* = 0.031) were independent predictors for PFS. Similarly, smoking history (HR, 0.440; 95% CI, 0.250–0.775; *p* = 0.004), stage Ⅳ (HR, 0.445; 95% CI, 0.209–0.947; *p* = 0.036), NLR (HR, 1.283; 95% CI, 1.120–1.469; *p* < 0.001), MCH (HR, 0.852; 95% CI, 0.752–0.965; *p* = 0.012), and RDW (HR, 1.044; 95% CI, 1.001–1.091; *p* = 0.042) independently predicted OS.

**TABLE 2:** Cox analyses for progression-free survival and overall survival.

Variables	Progression-free survival				Overall survival			
	Univariable		Multivariable		Univariable		Multivariable	
	HR (95% CI)	*p* value	HR (95% CI)	*p* value	HR (95% CI)	*p* value	HR (95% CI)	*p* value
Age	0.998 (0.980–1.018)	0.872			1.001 (0.974–1.030)	0.917		
Sex (Male)	0.649 (0.407–1.036)	0.070			0.613 (0.329–1.141)	0.123		
Smoking history (Ever)	0.562 (0.384–0.822)	0.003	0.457 (0.306–0.683)	< 0.001	0.627 (0.369–1.066)	0.085	0.440 (0.250–0.775)	0.004
ECOG PS (≥1)	1.796 (0.870–3.709)	0.113	3.040 (1.386–6.668)	0.006	1.474 (0.583–3.731)	0.413		
Stage (Ⅳ)	0.664 (0.399–1.105)	0.115	0.465 (0.267–0.812)	0.007	0.698 (0.340–1.433)	0.327	0.445 (0.209–0.947)	0.036
Histology (SCC)	0.687 (0.470–1.005)	0.053			0.689 (0.406–1.169)	0.167		
PLR	1.001 (1.000–1.003)	0.135			1.002 (1.000–1.004)	0.040		
NLR	1.077 (1.012–1.147)	0.020	1.766 (1.311–2.380)	< 0.001	1.162 (1.076–1.255)	< 0.001	1.283 (1.120–1.469)	< 0.001
dNLR	1.023 (0.935–1.121)	0.617	0.489 (0.321–0.744)	0.001	1.127 (1.008–1.260)	0.035	0.823 (0.650–1.041)	0.103
MLR	2.526 (1.433–4.452)	0.001	0.203 (0.044–0.929)	0.040	2.845 (1.392–5.816)	0.004		
SII	1.000 (1.000–1.000)	0.060			1.000 (1.000–1.001)	< 0.001		
HGB	0.990 (0.979–1.000)	0.053	0.002 (0.001–0.171)	0.010	0.980 (0.966–0.994)	0.005		
RBC	0.807 (0.593–1.099)	0.173			0.663 (0.434–1.012)	0.057		
WBC	1.029 (0.970–1.093)	0.342			1.070 (0.994–1.153)	0.073		
NEUT%	1.007 (0.991–1.022)	0.403			1.023 (1.000–1.047)	0.053		
LYM%	0.978 (0.958–0.999)	0.044			0.956 (0.926–0.987)	0.006		
MONO%	1.017 (0.966–1.071)	0.528			0.971 (0.897–1.051)	0.466		
EOS%	1.043 (0.967–1.126)	0.271			0.950 (0.837–1.077)	0.421		
BASO%	0.611 (0.297–1.258)	0.181			0.349 (0.086–1.413)	0.140		
PLT	1.000 (0.998–1.002)	0.773			1.001 (0.998–1.004)	0.512		
HCT	0.962 (0.927–0.998)	0.037	2.220 (1.183–4.166)	0.013	0.937 (0.893–0.983)	0.008		
MCV	0.968 (0.934–1.003)	0.069	0.678 (0.525–0.876)	0.003	0.961 (0.918–1.007)	0.093		
MCH	0.911 (0.824–1.008)	0.072			0.853 (0.759–0.960)	0.008	0.852 (0.752–0.965)	0.012
MCHC	0.999 (0.980–1.018)	0.894	1.020 (0.999–1.041)	0.065	0.974 (0.949–0.998)	0.036		
RDW	1.016 (0.987–1.047)	0.282	1.052 (1.005–1.102)	0.031	1.038 (0.998–1.079)	0.064	1.044 (1.001–1.091)	0.042
PCT	0.998 (0.976–1.020)	0.844			1.010 (0.980–1.041)	0.509		
PDW	0.959 (0.864–1.064)	0.431			0.977 (0.845–1.129)	0.751	0.577 (0.308–1.080)	0.085
P-LCR	0.988 (0.964–1.012)	0.333			1.002 (0.969–1.036)	0.915	1.148 (0.993–1.327)	0.062
CEA	0.796 (0.386–1.634)	0.533			0.865 (0.367–2.130)	0.755		

ECOG PS, Eastern Cooperative Oncology Group Performance Status; SCC, squamous cell carcinoma; PLR, platelet-to-lymphocyte ratio; NLR, neutrophil-to-lymphocyte ratio; MLR, monocyte-to-lymphocyte ratio; SII, systemic immune-inflammation index; HGB, hemoglobin; RBC, red blood cell count; WBC, white blood cell count; NEUT, neutrophil; LYM, lymphocyte; MONO, monocyte; EOS, eosinophils; BASO, basophil granulocytes; PLT, platelet; HCT, hematocrit; MCV, mean corpuscular volume; MCH, mean corpuscular hemoglobin; MCHC, mean corpuscular hemoglobin concentration; RDW, red blood cell distribution width; PCT, plateletcrit; PDW, platelet distribution width; P-LCR, platelet-larger cell ratio; CEA, carcinoembryonic antigen; HR, hazard ratio; CI, confidence interval.

**FIGURE 2:**
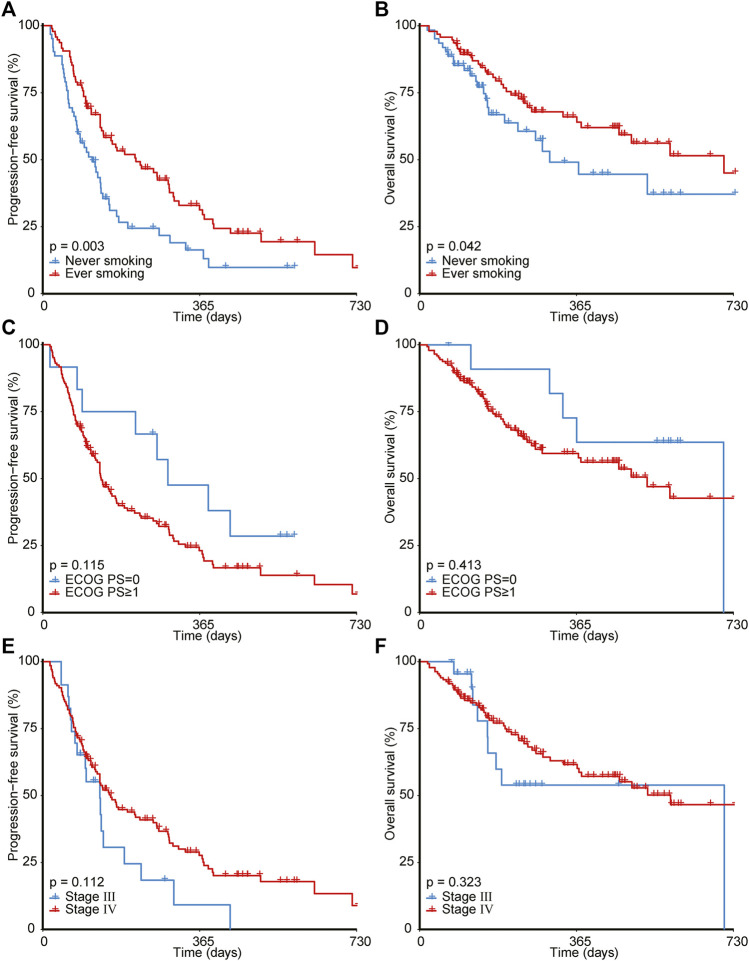
Survival analyses for patients with different smoking history **(A,B)**, ECOG PS **(C,D)**, and tumor stage **(E,F)**. ECOG PS, Eastern Cooperative Oncology Group Performance Status.

**FIGURE 3:**
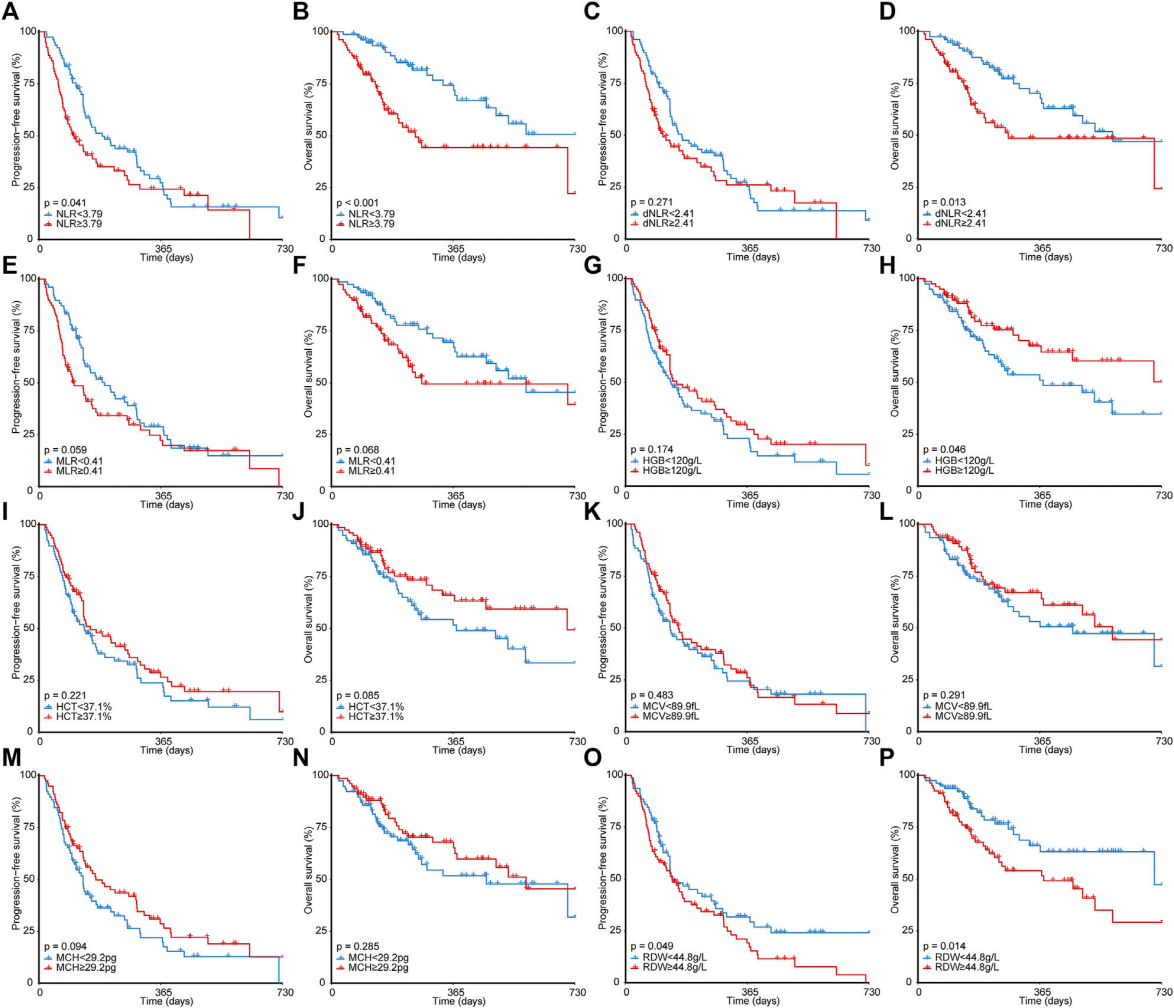
Survival analyses for patients with different NLR **(A,B)**, dNLR **(C,D)**, MLR **(E,F)**, HGB **(G,H)**, HCT **(I,J)**, MCV **(K,L)**, MCH **(M,N)**, and RDW **(O,P)**. NLR, neutrophil-to-lymphocyte ratio; MLR, monocyte-to-lymphocyte ratio; HGB, hemoglobin; HCT, hematocrit; MCV, mean corpuscular volume; MCH, mean corpuscular hemoglobin; RDW, red blood cell distribution width.

As illustrated in Figure 2, ever-smoking patients achieved significantly better PFS (1-year PFS, 31.2% [95% CI, 22.2%–43.9%] *versus* 16.3% [95% CI, 8.3%–31.7%], *p* = 0.003) and OS (1-year OS, 64.0% [95% CI, 53.8%–76.3%] *versus* 49.1% [95% CI, 34.8%–69.1%], *p* = 0.042) compared with never-smoking patients. However, ECOG PS and stage failed to stratify the prognosis after immunotherapy. Moreover, as shown in Figure 3, by utilizing the median value as the cut-off, NLR ≥3.79 (1-year PFS, 24.2% [95% CI, 15.2%–38.4%] *versus* 27.3% [95% CI, 18.2%–41.1%], *p* = 0.041; 1-year OS, 44.2% [95% CI, 32.5%–60.1%] *versus* 71.8% [95% CI, 60.6%–85.2%], *p* < 0.001) or RDW ≥44.8 g/L (1-year PFS, 19.2% [95% CI, 11.4%–32.3%] *versus* 31.7% [95% CI, 21.9%–46.0%], *p* = 0.049; 1-year OS, 54.0% [95% CI, 42.7%–68.3%] *versus* 63.1% [95% CI, 50.6%–78.6%], *p* = 0.014) was significantly correlated to poorer PFS and OS than NLR< 3.79 or RDW< 44.8 g/L. In addition, patients with dNLR ≥2.41 (1-year OS, 48.5% [95% CI, 36.6%–64.4%] *versus* 67.8% [95% CI, 56.3%–81.5%], *p* = 0.013) or HGB <120 g/L (1-year OS, 51.1% [95% CI, 39.6%–65.9%] *versus* 67.6% [95% CI, 56.0%–81.7%], *p* = 0.046) showed inferiority only in OS than those with dNLR< 2.41 or HGB ≥120 g/L. However, other blood biochemical parameters did not stratify the prognosis of NSCLC receiving immunotherapy.

### Predictive performance of peripheral blood markers

**FIGURE 4:**
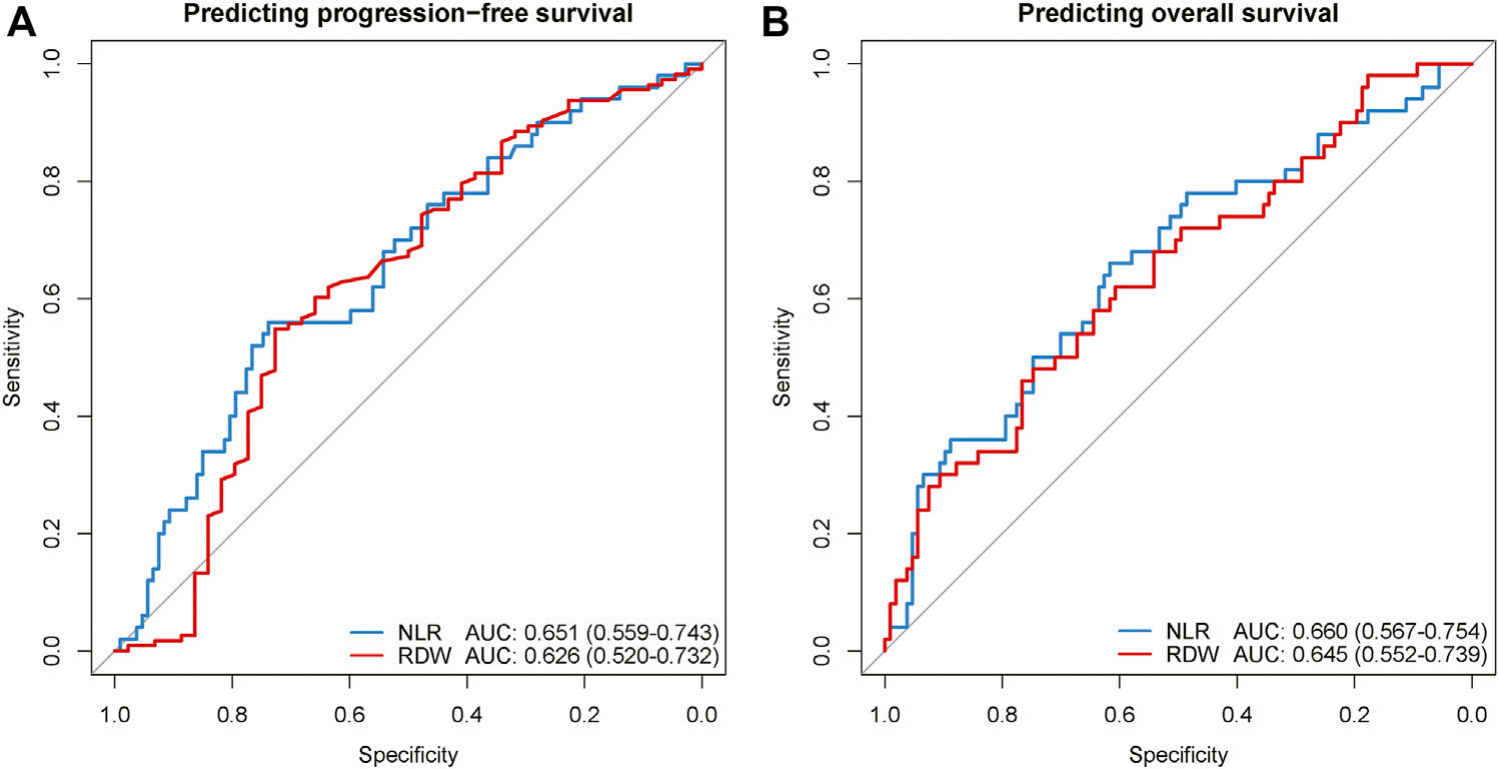
The ROC curves of NLR and RDW for predicting regression-free survival **(A)** and overall survival **(B)**. ROC, receiver operating characteristic; AUC, area under the curve of the receiver operating characteristic; NLR, neutrophil-to-lymphocyte ratio; RDW, red blood cell distribution width.

Considering PLR and RDW were two independent inflammatory biomarkers for both PFS and OS, the receiver operating characteristic analysis was implemented to quantify the predictive performance of PLR and RDW (Figure 4). For predicting PFS, NLR and RDW achieved areas under the curves (AUCs) with 0.651 (95% CI, 0.559–0.743) and 0.626 (95% CI, 0.520–0.732). Similarly, in the prediction for OS, the performances of NLR and RDW were shown to have AUCs of 0.660 (95% CI, 0.567–0.754) and 0.645 (95% CI, 0.552–0.739).

## Discussion

Despite immunotherapy having revolutionized the treatment paradigms of NSCLC (Okazaki et al., 2013; Ribas and Wolchok, 2018), the low response rate, therapy-related adverse effects, and high medical expense emphasize the significance of biomarkers for immunotherapeutic efficacy (Borghaei et al., 2015; Brahmer et al., 2015; Reck et al., 2016). In this study based on a multicenter population, we demonstrated that higher NLR and RDW in baseline peripheral blood were significantly correlated with poor PFS and OS in NSCLC patients undergoing ICIs treatment.

Previously, a number of studies have made investigations on this topic and revealed that TMB, PD-L1, and tumor-infiltrating lymphocytes derived from core biopsy specimens were correlated with immunotherapy prognosis of NSCLC (Kerr et al., 2015; Meng et al., 2015; High TMB Predicts Immunotherapy Benefit, 2018). However, these biomarkers suffered from biopsy-related morbidities due to their invasive nature. To overcome this limitation, further studies found that these markers in the peripheral blood also hold the potential to predict immunotherapy efficacy (Gandara et al., 2018; Wang et al., 2019; Bratman et al., 2020). Despite this breakthrough, these blood biomarkers were quantified based on peripheral blood mononuclear cells, which are too costly and time-consuming to acquire. In contrast, peripheral blood markers derived from routine complete blood count (CBC) are easily accessible and cost-effective, and thereby could be utilized as a convenient instrument in routine clinical practice.

Findings in our study were in line with previous publications that higher NLR was an adverse factor for the prognosis of NSCLC receiving immunotherapy (Fukui et al., 2019; Valero et al., 2021). In addition, Diem et al. (2017) concluded that PLR also played an important role in predicting immunotherapy response and prognosis and NSCLC patients with higher PLR tended to have an inferior prognosis. However, our study failed to validate the predictive efficiency of PLR: we speculated it might be attributable to that our study also included other biochemical parameters in the routine peripheral blood examination. Interestingly, we proved that increment of RDW significantly predicted poorer PFS and OS in NSCLC treated by immunotherapy, which was also observed in diffuse large B-cell lymphoma receiving immunotherapy (Beltran et al., 2019), but limited previous studies demonstrated its value in the NSCLC population. As such, we first indicated the capability of RDW as the biomarker for immunotherapeutic efficacy, and this finding might imply further insight into the prediction of immunotherapy response.

In addition to clinical implications, it is important to understand the biological basis underlying the prediction of NLR and RDW. The predictive mechanism of NLR might be rooted in its contributions to an immunosuppressive tumor microenvironment. On the one hand, as neutrophils were capable of releasing components mediating immunosuppression and tumor angiogenesis, neutrophil infiltration, thereby, established a microenvironment promoting cancer initiation, proliferation, and metastasis (Gonzalez et al., 2018; Shaul and Fridlender, 2019). On the other hand, reduced densities of lymphocyte infiltration contributed to the decreased response of antitumor T-cell, and the high level of neutrophils might further restrain T-cell response (Restifo et al., 2012; Zito Marino et al., 2017).

RDW, as an indicator representing the variations in the shape and size of red blood cells, is easily accessible in a routine CBC examination. The increased level of RDW implies a sign of impairments in erythropoiesis and red blood cell metabolism. The mechanism underlying the correlation of RDW with immunotherapeutic efficacy has not been clarified. However, several publications revealed that increasing RDW might result from oxidative stress, inflammation, and poor nutritional status *via* variation of erythropoiesis (Salvagno et al., 2015), and emerging pieces of evidence indicate that RDW was an adverse predictor for the prognosis of multiple malignancies (Koma et al., 2013; Albayrak et al., 2014; Ay et al., 2015).

Still, several limitations existed in the current study. First, despite the inclusion of a multicenter population, this study was limited by its retrospective nature, which suffered from selection bias and potential confounders. We utilized the multivariable regression to adjust prognostic predictors, but the impact of some known biomarkers, such as TMB, could not be evaluated. Thus, future prospective studies are required to validate our conclusions. Second, the small sample size reduces the power of the current study, and to be confirmed, further follow‐up studies enrolling a larger sample size need to be performed. Finally, the underlying mechanism of the biomarkers has not been elucidated, and future studies focusing on the biological basis of NLR and RDW are warranted.

## Conclusion

Our study demonstrated that NLR and RDW in baseline peripheral blood could help stratify the prognosis of advanced NSCLC patients receiving immunotherapy. Thus, NLR and RDW harbor the potential to serve as effective biomarkers for immunotherapeutic efficacy in NSCLC.

## Data Availability

The original contributions presented in the study are included in the article/Supplementary Material; further inquiries can be directed to the corresponding author.
